# Skeletons, Object Shape, Statistics

**DOI:** 10.3389/fcomp.2022.842637

**Published:** 2022-10-18

**Authors:** Stephen M. Pizer, J. S. Marron, James N. Damon, Jared Vicory, Akash Krishna, Zhiyuan Liu, Mohsen Taheri

**Affiliations:** 1 Department of Computer Science, University of North Carolina at Chapel Hill, Chapel Hill, NC, United States; 2 Department of Statistics and Operations Research, University of North Carolina at Chapel Hill, Chapel Hill, NC, United States; 3 Department of Mathematics, University of North Carolina at Chapel Hill, Chapel Hill, NC, United States; 4 Kitware Inc., Carrboro, NC, United States; 5 Department of Mathematics and Physics, University of Stavanger, Stavanger, Norway

**Keywords:** shape, skeleton, shape statistics, skeletal model, s-reps

## Abstract

Objects and object complexes in 3D, as well as those in 2D, have many possible representations. Among them skeletal representations have special advantages and some limitations. For the special form of skeletal representation called “s-reps,” these advantages include strong suitability for representing slabular object populations and statistical applications on these populations. Accomplishing these statistical applications is best if one recognizes that s-reps live on a curved shape space. Here we will lay out the definition of s-reps, their advantages and limitations, their mathematical properties, methods for fitting s-reps to single- and multi-object boundaries, methods for measuring the statistics of these object and multi-object representations, and examples of such applications involving statistics. While the basic theory, ideas, and programs for the methods are described in this paper and while many applications with evaluations have been produced, there remain many interesting open opportunities for research on comparisons to other shape representations, new areas of application and further methodological developments, many of which are explicitly discussed here.

## INTRODUCTION

**FIGURE 1 ∣:**
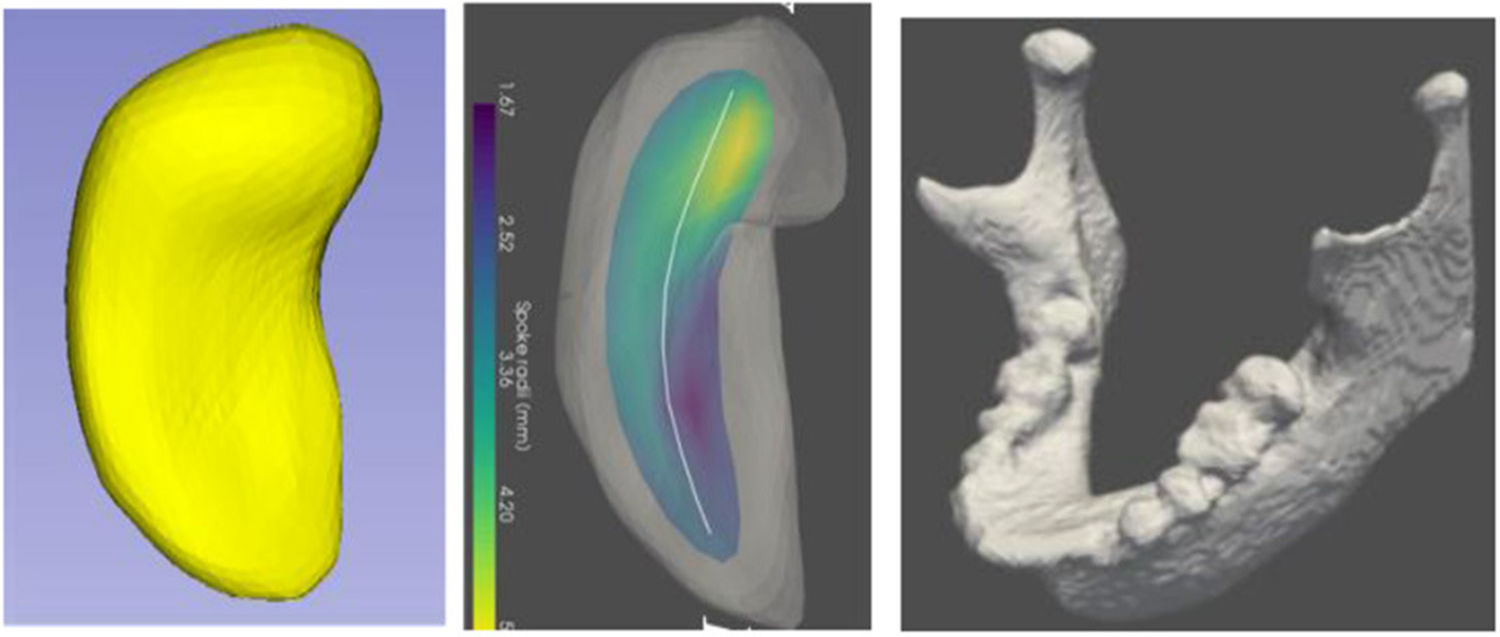
Left: A hippocampus, a C-shaped object. Middle: the hippocampus with its skeletal surface (colored, with the color indicating the width of the object there) and its spine (the curve that is its long axis). Right; a mandible, another C-shaped object.

This paper discusses models of objects, in 3D and 2D but with special emphasis on 3D. The concern is with both individual objects and complexes of multiple objects and, especially, *objects and complexes that appear as populations of instances*. The objects of concern have curved slab-like shapes, with no branching or a limited, fixed amount of branching. I^1^ call these objects “slabular,” with tube-like objects, i.e., generalized cylinders, being a special case of slabular objects. The examples we will start with are anatomic objects, such as the hippocampus or mandible (jaw-bone) shown in Figure 1^2^, but as will be seen later, the ideas apply to many manufactured objects, such as shoe boxes or airplanes.

The purpose of this paper is to survey a few decades of work on a skeletal model called “s-reps,” designed for statistics. The paper provides many mathematical and statistical concepts, definitions, and properties. It describes many algorithms related to forming and using s-reps, and it describes many uses to which they have been put. It argues that the focus of these models on the objects themselves and capabilities of s-reps in richly capturing geometric properties, especially for statistical applications, make it an important way of describing shape.

The most common alternative approaches to representing shape are point distribution models (PDMs), especially focusing on boundary points, and models describing shape via diffeomorphisms from a containing space enclosing the objects (e.g., Durrleman et al., 2014), as distinct from s-rep methods described here that are formed by diffeomorphisms of object interiors. The experimental data to date, cited and/or presented in this paper is that s-reps are superior to PDMs for statistical applications and that using s-reps to form PDMs has superiority to other approaches of forming PDMs. We are not aware of experiments comparing s-reps to the methods based on diffeomorphisms of a containing space for statistical purposes, and **we look forward to such comparisons**.

Despite decades of development, in laboratories at or in collaboration with mine at UNC, there are many statistical, mathematical, algorthmic, and applications challenges still to be met, as well as further studies comparing s-reps to other shape representations. **One of the objectives of this paper is to lay out these remaining challenges and opportunities: each of these are indicated in the Bold font**.

For a slabular object there exists a smooth sequence of usually non-parallel slicing planes such that no successive planes intersect within the object and such that the object boundary’s intersection with each plane is eccentric (one of the two principal axes’ radii is notably longer than the other). As described in Section Skeletal Models and S-reps: Definitions and Mathematics, the locus of centers of the cross-sections forms a curvilinear axis, which I call the “spine,” that is notably longer than the axes in the cross-sections. For example, for the hippocampus seen in Figure 1 the long axis is C-shaped and goes from the tip (at the top of the figure) to the tail, the short axis goes from the front of the object as seen in Figure 1 left to the back from that point of view, and the middle-length dimension goes from side to side as seen in that Figure. In the mandible the shortest axis goes from the facial side to the inside of the mouth, the longest axis goes from one temporal-mandibular joint (TMJ, where the mandible hinges on the skull) to the other, and the middle-length axis goes from the teeth positions extended to the TMJs down to the chin locus extended to the TMJs. If the longest axis terminates in the two knob-shaped entities (called the “condyles”), the pointy figures opposite the condyles, called the “coronoid processes,” form subfigures. These subfigures can be found in essentially every human mandible.

Since all slabular objects have a central curve, formed by the spine, and cross-sections, they can all be understood as generalized cylinders. However, when the cross-sections’ two axes are not too different in length, that is, the cross-sections are not too far from circular, the generalized cylinder is more tube-like.

**FIGURE 2 ∣:**
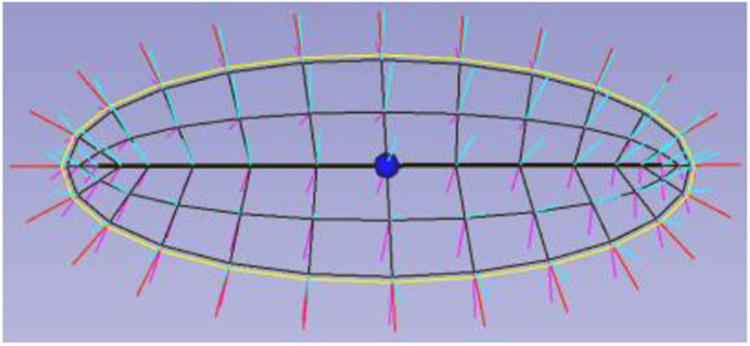
An ellipsoid’s skeleton sampled into a grid and its spokes; spokes at the skeletal fold are displayed as red, those on the north side of the skeleton in cyan, and those on the south side in magenta. The spine is bold, and the center point is displayed as a bullet.

While the most common computer representations of such objects capture either their boundary locations alone or deformations of the whole ambient space in which the objects reside, it has been seen by many (e.g., Blum and Nagel, 1978; Amenta and Choi, 2008; Siddiqi, 2008; Székely, 2008; Yushkevich et al., 2015) that object widths, which are captured by the shorter cross-sectional axes, are important features as are features derivable from the behaviors of specified directions, especially boundary normals (Srivastava et al., 2011). Since these features are exactly what skeletal models capture, in quite a variety of applications such models have been shown more powerful than those based on the other types of object representations. From the point of view of slabular objects the skeleton (Figure 1, middle) includes the spine as its long axis, and the cross-sectional dimensions can be captured by line segments emanating at the spine and ending at the edge of the skeleton (Figure 2). We call these line segments “spokes”.

Given a population of objects skeletally represented, it is useful to model the populations via probability densities. It has been shown that these can be of use for objectives such as classification into subpopulations, hypothesis testing on differences between populations, and segmentation approaches that use shape prior distributions as well as image intensity features understood via the spatial correspondences that the geometric model provides. In this paper both a particularly effective form of skeletal model called “s-reps” will be motivated and described, and methods of computationally deriving s-reps from object boundaries will be overviewed. In statistical applications, s-reps can provide an effective way to establish locational and orientational correspondences across the shape samples in the population. In this paper, not only the s-reps but also means of accomplishing the statistical objectives with respect to s-reps will be covered.

Many examples of applications of s-reps and complexes thereof, especially in medical-imaging-based data, will be given. However, the intent here is also to stimulate applications in many other areas of computer analysis and synthesis, including those on manufactured objects.

## SKELETAL MODELS AND S-REPS: DEFINITIONS AND MATHEMATICS

**FIGURE 3 ∣:**
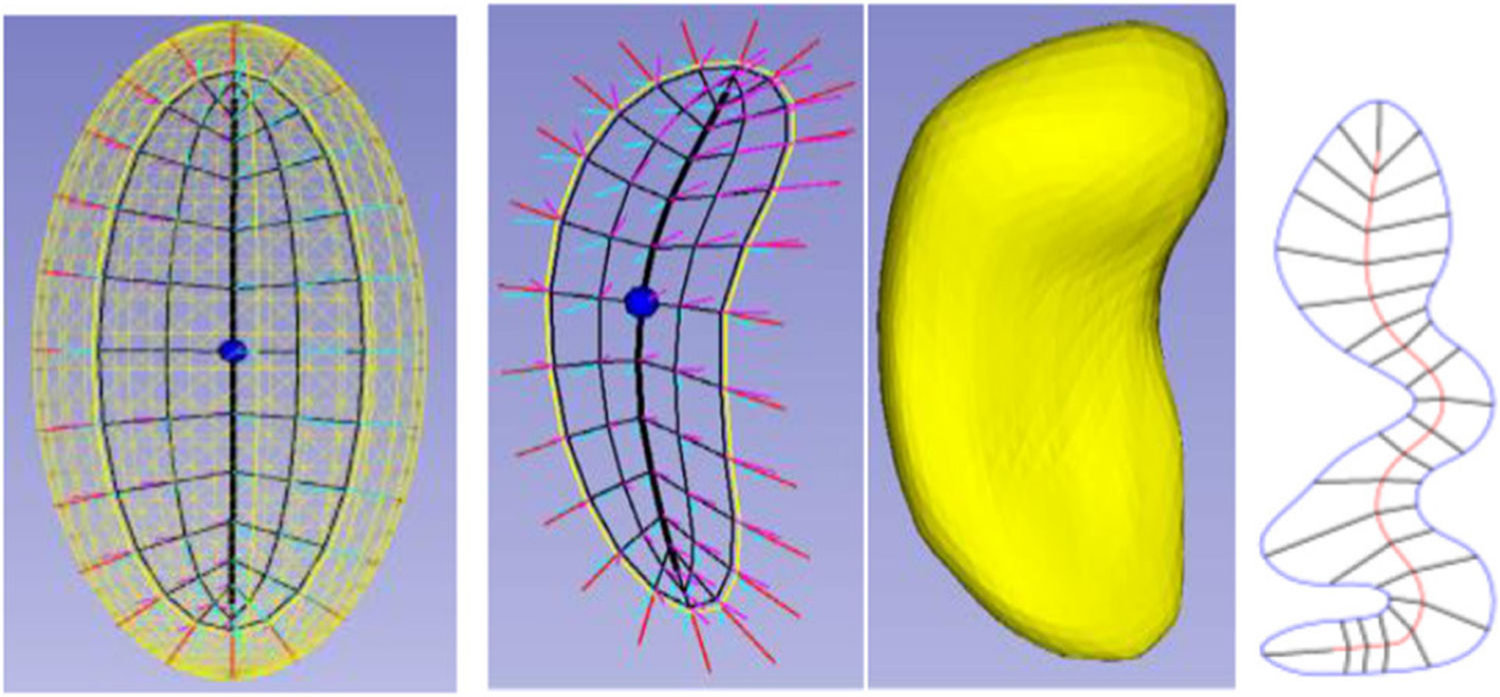
Skeletal models. Left: Ellipsoid (3D) with its elliptical skeleton (grid), spine (bold), and center point (bullet). Middle left: A skeleton and its spokes for the hippocampus (3D) shown in middle right. Only samples of the spoke vectors are shown, but they exist continuously, i.e., with their tails at every continuous point of the skeletal surface. Right: A skeleton (red) and its spokes for a 2D object.

**FIGURE 4 ∣:**
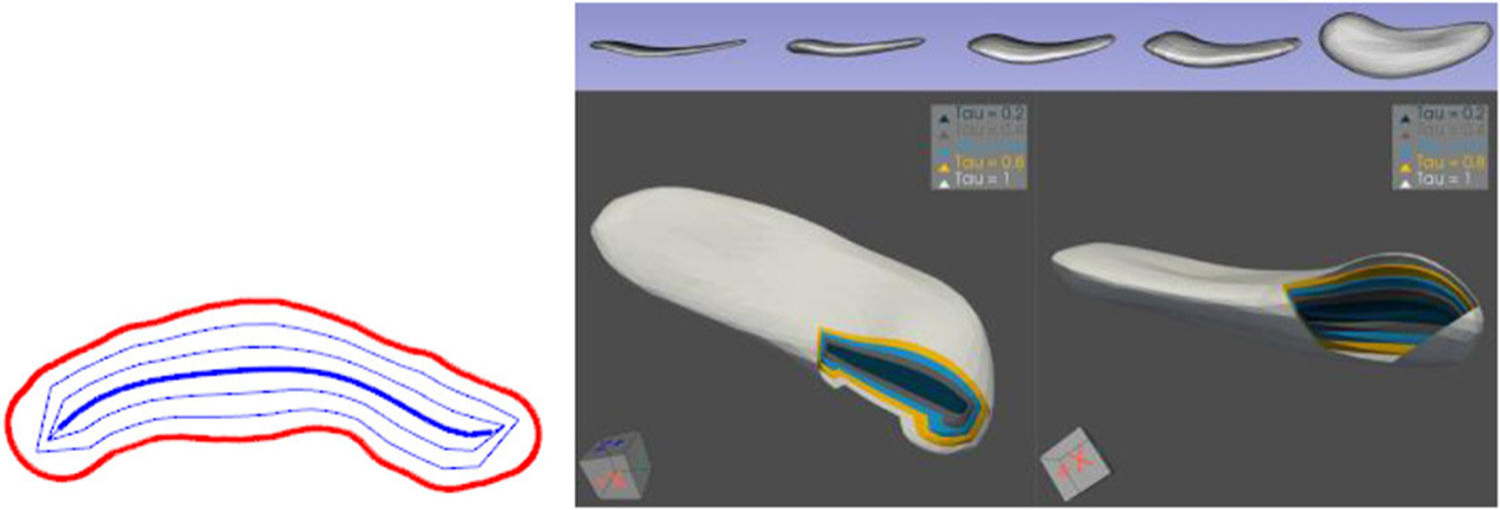
Left: Radial onion skins in 2D. Right: Onion skins at τ_2_ = 0.0, 0.2, 0.4, 0.6, 0.8, and 1.0 (boundary) for a hippocampus; top row: individual onion skins for the various τ_2_ values. Bottom row: two views of those onion skins seen end-on for a cut through the object.

Roughly speaking, a skeletal model of an object (Figures 1-3) consists of a) a skeletal locus somehow central along the object and b) spokes, vectors emanating from the skeleton and ending at the object boundary, such that the spokes do not cross within the object. Traditionally, the skeleton has been considered a possibly branching surface or curve, but I find it much clearer to consider it as a collocated pair of surfaces or curves formed by a sort of collapse of the object boundary or better, which can be dilated to form the boundary implied by the skeleton and its spokes (Figure 4). In this way of thinking, the skeleton shares topology with the object boundary; it just has the constraint that except along the curve where the skeleton folds, the two copies of the skeletal surface or curve share the same skeletal spoke end point. This point of view allows the possibilities of having different spoke statistics for one of the skeletal copies than the other. Later in this paper, I will cover possibilities of relaxing the constraint of being a perfect skeletal copy in certain segments of the skeleton.

The history and mathematics of skeletal models, as well as a number of algorithms for extracting them from object boundaries, is covered in detail in the book by Siddiqi (2008). Harry Blum, the inventor of the earliest form of skeletal model (Blum and Nagel, 1978), which we now call the “Blum medial axis” felt that the major strength of medial models was that it provided a subdivision of an object into various attached parts. This goal turned out to be unachievable due the extreme bushiness: deep, broad, and random branching of the Blum medial axis; this bushiness was a consequence of the inevitable noise in the object boundary—this will be discussed at length shortly. However, the facts that the spoke lengths were a measure of object (half-)width and that the spoke directions and their derivatives were important indications of local object orientation and curvature have turned out to be particularly powerful measurements in object representation.

Blum, an inspired engineer, defined the Blum medial axis (that he called the “symmetric axis”) in terms of a flow at a constant rate (in the Euclidean metric in the object’s ambient space). He called this flow by the word “grassfire” collapsing the object boundary to the medial axis. Blum and Nagel (1978) developed some early mathematics of this mapping from boundary to this skeletal structure, and many mathematicians took up the challenge of providing further properties; of special interest was the definition of a generalization they called the “symmetry set.” In it the spokes were always orthogonal to the object boundary, a property Damon calls “partial Blum.” Thus, it captured the boundary normals. The work of Srivastava et al. (2011) and others has emphasized that the behavior of these boundary normals is an important characteristic of shape. The way that they swing as you move along the boundary is the curvature information promulgated by Gauss (Koenderink, 1990).

The study of the symmetry set culminated in its singularity theoretic analysis by Giblin et al., a beautiful summary of which appears in Giblin and Kimia (2008). This work led to the important understanding that branching of the Blum skeleton was generic in both 2D and 3D.

The experience of hundreds of authors attempting to produce algorithms to map the object boundary to the Blum medial axis yielded an understanding that small protrusions or indentations in the boundary, either real or produced by noise, led to an extraordinary bushiness of branching and that the bush had to be heavily pruned if the skeletal structure was to be of any use—but especially in 3D robust algorithms to do this pruning were elusive. Certainly, little success in providing statistics on these derived structures was achieved due to the variation of this branching structure over a population of shapes.

The group I have led concluded that the branching structure needed to be fixed in order to support statistics. Supporting this need, my colleague James Damon invented a “reverse” type of flow, which he called “radial flow” (Figure 4), from the skeleton to a close approximation of the boundary. In it the skeleton, given its spokes, flowed (dilated) at a rate proportional to the spoke length. That is, for a spoke of length *r*, positions along the spoke were considered as *τ* r, where *τ* = 0.0 on the skeleton, *τ* = 1.0 on the object boundary, and *τ* = 0.5 halfway from the spoke end on the skeleton and the spoke end on the boundary. Damon (2008), and in more mathematical detail (Damon, 2003, 2004), discovered the mathematics of radial flow and the idea of the “onion-skin” surface at any given radial distance *τ* from the skeleton; a particular accomplishment was his invention of a matrix-valued function on the skeleton, S_rad_ that yielded the velocity at which the spokes swung as you moved along the skeleton and a pair of eigenvalues called radial curvatures, whose comparisons to the corresponding boundary curvatures allow preventing spokes from crossing in the interior of the object. All this applied not only to the Blum skeleton but also to Damon’s generalization that did not need to meet all of the Blum properties. Among other things, the two spokes with collocated tails did not need to be of equal length and they did not need to be orthogonal to the boundary (be “partial Blum”). In order for the entity to capture width and direction properties, I added soft restrictions that this axis should rather closely achieve the properties of the Blum medial axis (Pizer et al., 2013; Liu et al., 2021a), and I named this form of skeletal structure the “s-rep.” S-reps are in this generalized skeletal category; almost always they are not Blum-medial and are designed to support statistical analysis.

The design was that a discretized form of the s-rep would be fit to the boundary, avoiding the problem of bushiness. As described and discussed in the next section, my colleagues and I produced software that by such fitting yielded the s-rep whose thus-dilated skeleton well fit the object boundary in both 2D and 3D and would with some tolerance meet the partial-Blum objectives.

For multiple objects, the shape information should include not only the shape of the individual objects but also their geometric relationships. Blum and Nagel had already described the “external medial axis,” which is the Blum medial axis of the complement of the objects. However, as pointed out by Damon and Gasparovic (2017), this axis was necessarily branching for 3 or more objects, even if the objects’ skeletons had consistent branching. But using radial flow past the objects’ boundaries, Damon extended the flow to what he called a linking axis, which typically branches, and as described in Section Multi-object Statistics Using Skeletal and Boundary Fitted Frames, Liu showed how to produce such a flow that does not branch.

After some experimentation with representations that included object angle, which were rejected because the bisector of the object angle was ambiguous when the angle was π/2, the UNC team settled on a representation by the spoke skeletal location, the spoke direction, and the spoke length (object’s local half-width) on the skeletal grid positions, since these emphasized the properties on which statistics could best be focused, we thought. Some applications represented a spoke by the coordinates of its two endpoints (skeletal and boundary).

**FIGURE 5 ∣:**
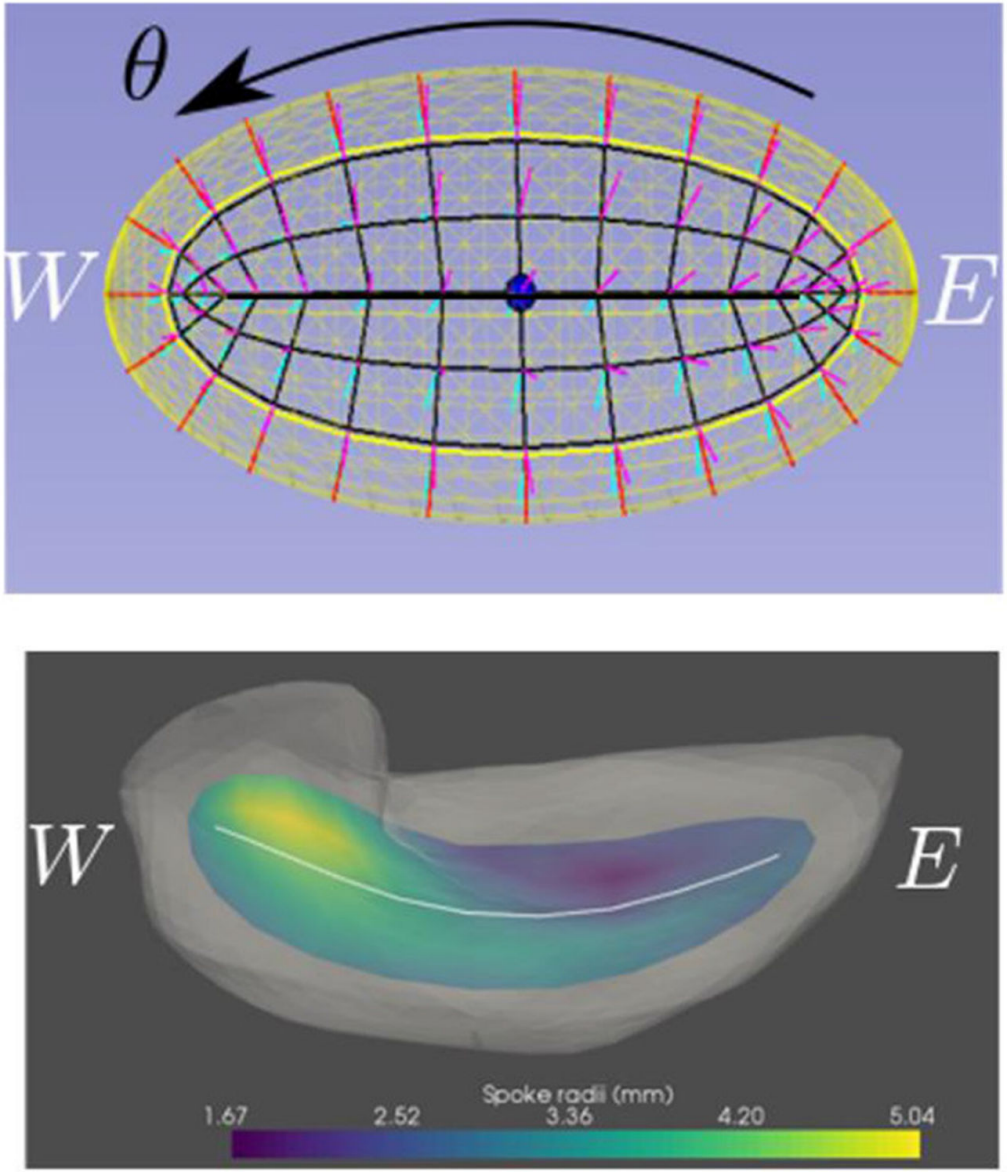
Top: The three parameters for an ellipsoid, as seen from a direction called “north”. The bold line is its spine; the bullet is its center point; the inner yellow curve is the fold of the skeleton; *θ* goes around the elliptical and quasi-elliptical curves; τ_1_ goes along the black, non-bold lines; τ_2_ goes from the skeletal plane out to the ellipsoid boundary. Bottom: the skeleton and spine transferred to the hippocampus.

I have come to see slabular objects as diffeomorphically deformed versions of the most basic slabular object, the (3D) ellipsoid. This view makes it analogous to the methods of shape representation via diffeomorphisms of a base object, but here the diffeomorphism is specifically on the object boundary and interior and we can take particular advantages of the inherent parameters of the skeletal positions for an ellipsoid (Figure 5) to produce correspondences needed for statistics.

Let us first understand a 2D medial representation of an ellipse. That (2D) ellipse, which is the medial surface for an ellipsoid, can be represented by *its* skeleton, which we call the object’s “spine,” made from a (1D) folded line, parameterized within its interior by the cyclic value *θ ϵ [0,2π]*, together with spokes from that skeleton to the ellipse’s boundary. *θ* is taken to be 0 at the center of the “north” side of the spine and to be *π* at the center of the “south” side of the spine. The spokes of the ellipse are parameterized by its radial flow value *τ*_1_, which, except for the spine ends (*θ = π/2* or *−π/2*), where *τ*_1_ ∈ [0,1], we take it to be in (−1,1) with the sign indicating which side of the spine the spoke is and ∣*τ*_1_∣ indicating the radial distance from the spine. Moreover, the (1D) spine can be represented by its skeleton, a 0D entity, i.e., a point, which we call the object’s *center* point and choose it on the “north side” of the skeleton, i.e., to be at *θ = 0*.

In fitting the s-rep to a new object by a diffeomorphism of the ellipsoid, including its boundary, its skeleton, its skeletal spokes, and its 3D spokes parameterized by the fraction multiples *τ*_2_, these ellipsoid points are carried by a diffeomorphism (warp) to form that new object, i.e., its skeletally implied boundary, its skeleton, its spine, and its center point. The skeletal coordinates of the ellipsoid are carried to their corresponding points in the target object; the desire that they are also radial lengths of the target object is so far not strictly met. This is discussed further in Section Fitting S-reps to Single- and Multi-object Boundaries.

As described shortly, a discretization of this representation is used in the SlicerSALT online toolkit. An interesting object type is a generalized cylinder, which is a representation by a central (skeletal) curve and its cross-sections. In that case one can think of the ellipsoid for which that object is a diffeomorphism as being very eccentric, i.e., having one of its principal radii very much longer than the other two. In that situation the ordinary 3D skeletal analysis yields a spine, or its extension to the diffeomorphism of the endpoints of the ellipsoid, and this spine can be taken as the central curve. Moreover, the “cross-sections” are then defined by the skeleton parameterized by (*θ, τ*_1_) and (*θ* + π, *τ*_1_), where *θ* ∈ (−π/2, π/2).

Repeating the idea of the transformation from a 3D ellipsoid to its medial representation of a 2D folded ellipse, and thence from one of the ellipses to its medial representation of a 1D folded line, results in the further transformation from one of the lines to its 0D medial representation, a center point. When the ellipsoid is carried to a target object by an appropriate diffeomorphism, that center point is carried to a place within the object that can be taken as its center. This location, being guaranteed to be within and reasonably central in the object, is a far better representation than the object’s center of mass, which can even be outside of the object. Finally, this idea can be generalized to higher dimensional hyperellipsoids with its principal radii notably different than the others and sortable into an increasing sequence. The succession of 1-lower dimensional hyperellipsoids, by a medial description of the just higher dimensional ellipsoids would allow our skeletal ideas to be generalized to dimensions higher than 3.

**FIGURE 6 ∣:**
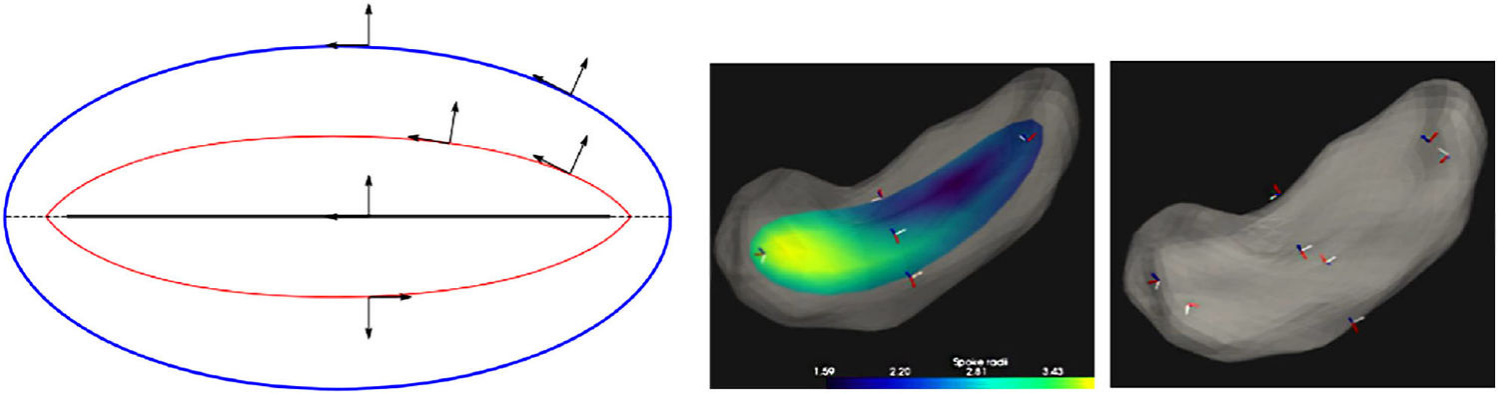
Fitted frames at various places: Left: Frames fitted to skeletal and boundary points on an ellipse in 2D. The solid, thicker, black line is the skeleton, here the spine; the dashed lines are the skeleton extension; the red curve is an onion skin; the blue curve is the ellipse boundary; arrows are local frames. Middle and Right: The hippocampus (gray); middle: with its s-rep’s skeleton (colored, with the color at a place on the skeleton showing the width of the object at that place) and fitted frames (blue element is normal) at the object center point and four points along and across the skeleton; right: the hippocampus boundary with fitted frames at points at ends of spokes at the skeletal points in the middle frame.

**FIGURE 7 ∣:**
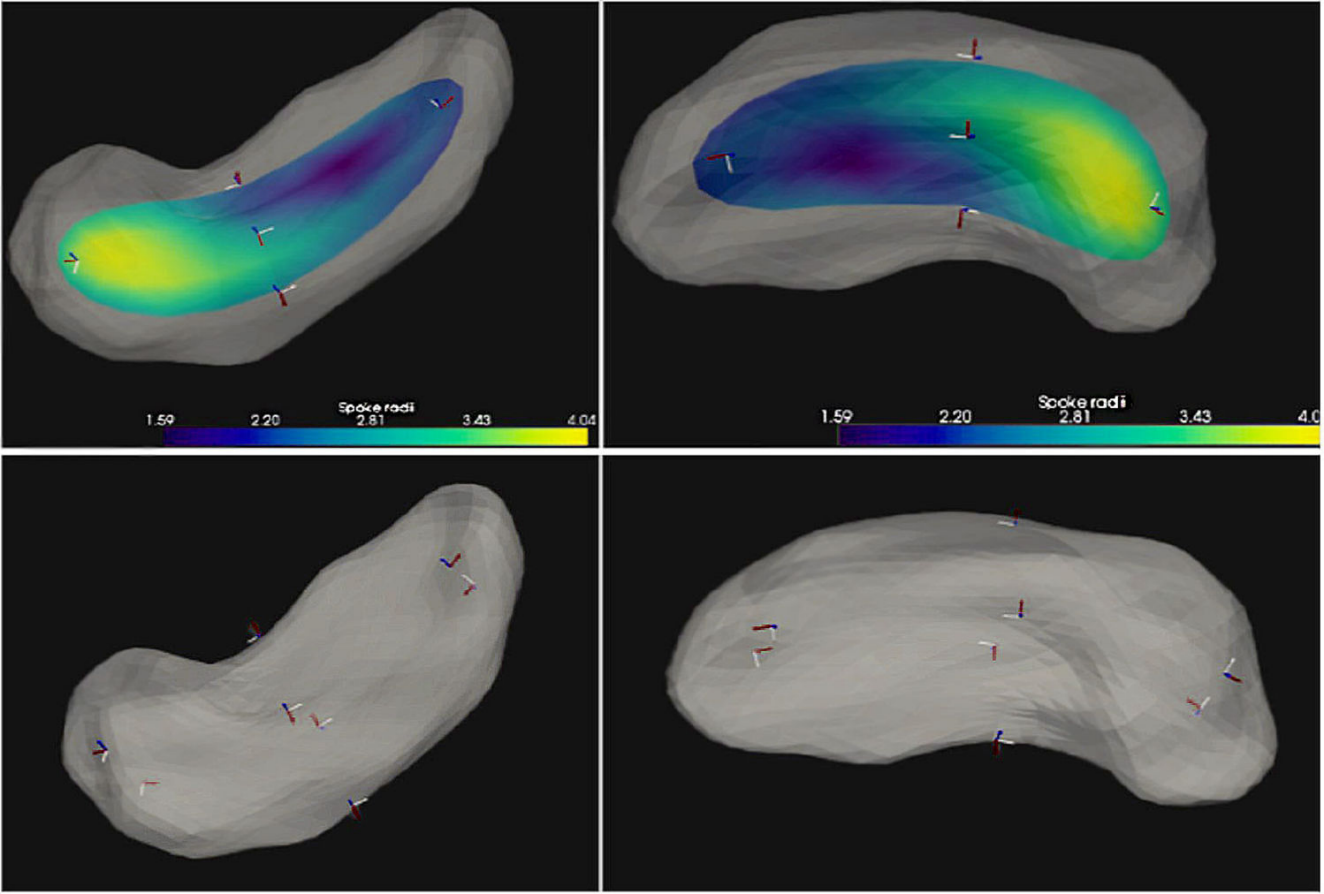
Skeleton and fitted frames for a hippocampus from two points of view. Upper 2 panels: frames on the skeletal locus, colored by object width, where blue the object is thin, and where yellow it is thicker. Five fitted frames are shown on the skeleton, with the normal shown in blue; their orientation carries information as to the curvature of the skeleton. Lower two panels: the hippocampus boundary with fitted frames at points at ends of spokes at the skeletal points in the middle frame.

Realizing that rotationally and translationally normalizing a set of s-reps was a difficult challenge, Taheri and Schulz (2021) took Cartan’s idea of representing space curves and surfaces using fitted frames and applied that idea to s-reps. Based on Taheri’s inspiration, I created the following structure for a fitted frame (see Figures 6, 7) (Pizer et al., 2021) that is consistent with skeletal geometry and with the fact that our s-reps are fit according to a diffeomorphism of an ellipsoid. It is based on the two radial flows just described, parameterized respectively by *τ*_1_ and *τ*_2_. Thus, any point inside the object represented by the s-rep is determined by a (*θ, τ*_1_, *τ*_2_) triple, each a function of Euclidean position within the object. If, as designed, common ellipsoid values of (*θ, τ*_1_, *τ*_2_), coming from diffeomorphisms from a common ellipsoid, yield statistically important correspondences, the fitted frames will yield statistically important orientation information.

The first vector of the fitted frame at any such point is taken to be tangent to the curve for the fixed (*τ*_1_, *τ*_2_) as the spine parameter *θ* varies, a direction tangent to the 3D onion-skin at parameter value *τ*_2_. The second fitted frame vector there is the normal to the *τ*_2_ onion-skin, with a sense away from the skeleton. Thus, on the spine of the skeleton (*τ*_1_ = 0, *τ*_2_ = 0) the fitted frame has vectors along the spine and orthogonal to the skeletal surface. Off the spine for *τ*_1_ >0 but on the skeleton, the fitted frame has one vector along the skeletal curve for *θ* varying and with fixed *τ*_1_, and a second vector orthogonal to the skeleton. On the boundary implied by the s-rep (for *τ*_2_ =1.0) at some *θ* and *τ*_1_, the first vector in the fitted frame is tangent to the implied boundary there, and the second vector in the fitted frame is normal to the implied boundary there.

Now, following Elie Cartan’s idea, geometric entities at a point should be understood according to the local fitted frame. The rotations of the fitted frames at any point interior to the object characterizes the local curvature of the object independent of rotations and translations of the object, which are important features. In Figure 6, for example, the rotation of the hippocampus skeleton from its center point to the end of the spine can be understood in terms of how the frame at the center point rotates into the frame at the end of the spine. Also, the rotation from the center point to its corresponding position on the boundary can be understood through the two frames at these positions. These rotations, as a function of three dimensions of motion, capture object curvature and can be fully characterized by three linear functions on a vector in 3-space (1-forms) measuring, respectively the rotations of the *τ*_2_-level surface normal (∇*τ*_2_) into the other two respective frame vectors in the tangent space and the rotation of one of those *τ*_2_-level surface frame vectors into the other. Not only these curvatures but also all other s-rep-relevant vectors when expressed in that fitted frame at the tail of the vector, are invariant to rotations and translations of the objects. Examples of such features, are the tangent to the spine, the skeletal spokes of the spine, and the 3D spoke directions at each sampled spine position and the vectors from each sample point to its neighbors in the ∇*τ*_1_ (along the 3D spokes), ∇*τ*_2_ (from the spine toward the skeletal fold), and ∇θ (along the spine) directions. Damon has pointed out that the fitted frames and the associated features depend on the diffeomorphism used from the ellipsoid to the object and thus are not inherent unless the choice of diffeomorphism is in some sense inherent to the object (see Section Fitting S-reps to Single- and Multi-object Boundaries).

Nevertheless, as presented in Section Statistics on S-reps for Single and Multiple Objects, Taheri and Schulz (2021) and Liu et al. (2021b) have shown serious advantages to classification and hypothesis testing when discretized features according to the fitted frame were used in the statistics.

## DISCRETE S-REPS

There have been two general ways suggested for discretizing a skeletal model. Yushkevich et al. (2003) did that by computing an appropriate spline, which discretizes by the discrete set of basis function coefficients defining the spline. In the work of my group the discretization is performed on each of the parameters representing the s-rep: *θ*, *τ*_1_, and *τ*_2_, into integer submultiples of their range, e.g., for the examples in Figures 2, 3, 5, *τ*_2_ as 0, ½, and 1; *τ*_1_ as—1, −½, 0, ½, 1 except at the spine ends, where its sampled values are 0, ½, 1; and *θ* into the cyclic values −π/2 for the east end of the spine, to π/2 for the west end of the spine, in steps of π/10 for the north side of the spine, and π/2 to 3π/2 in steps of π/10 for the south side of the spine. When the spokes are sampled only into 0 and 1, this produces a mesh of quadrilaterals on both the north and south sides of the skeleton, and corresponding meshes on the north and south sides of the object boundary (Figure 3, 2nd from left).

## MULTI-FIGURE S-REPS

**FIGURE 8 ∣:**
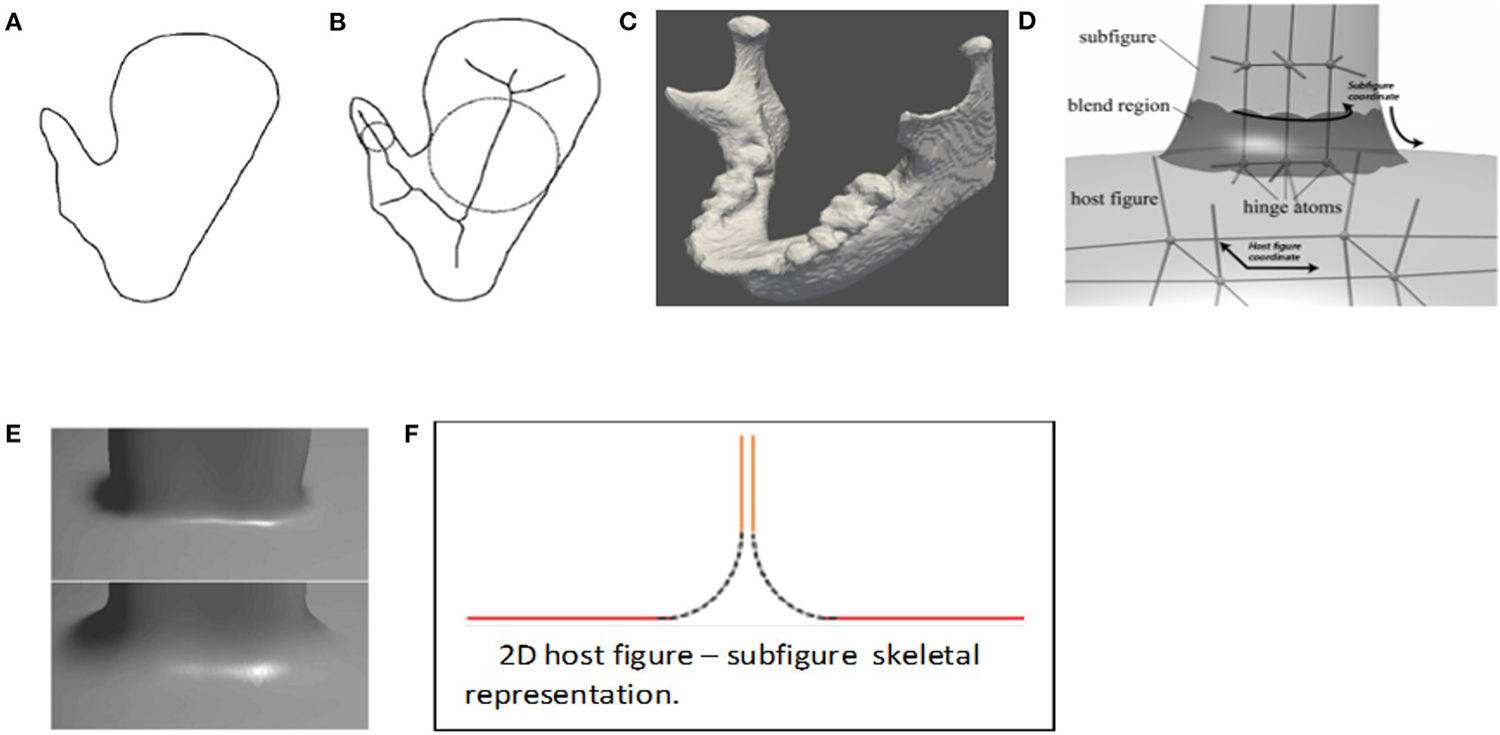
**(A)** A 2D figure with a protrusion. **(B)** The Blum representation of that object. **(C)** The mandible, with its protrusions. The **(D)** Han representation of object a [copied from Han et al., 2005]. The s-reps for the host figure and subfigure are attached by a skirt (darkened) connecting a cut through the subfigure to a one-sided hole in host figure skeleton. **(E)** At the figure, subfigure connection the boundary implied by **(D)**, for two different values of the blending parameter. **(F)** The skeletal arrangement for a host figure and subfigure, in 2D. The pairs of parallel lines are understood as collocated. The host figure is indicated by the red and blue lines; its bottom side (blue) has spokes facing downward, and its top side (red) has spokes facing upward. The top side has a hole in which the subfigure is fit. The subfigure skeleton (orange) is connected to the host figure skeleton by the skirt (dashed, black); together they have spokes facing right and left.

I use the term “figure” to refer to a geometric entity with an unbranching skeleton and which persists across a population of objects. An example is the coronoid process in the mandible (Figures 1, **mandible** and **10**). Traditionally, a population of objects in which there is a relatively sharp protrusion or indentation in similar positions along the boundary has been represented via the Blum medial axis (Figures 8A,B), i.e., with a branching skeleton. There are three difficulties with this representation: 1). It does not explicitly distinguish which two branches correspond to the host figure and which corresponds to the protrusion subfigure; 2). The host figure has a nonsmooth locus in its skeleton; 3). There is a long section of the skeleton of the protrusion branch that accounts for a very short part of the boundary. In regard to difficulty #1, for the 2D medial axis Katz and Pizer (2003) developed a method based on a model of human vision to distinguish the host skeleton from the subfigure skeleton, but this method is not mathematically characterized and has not been generalized to 3D. A method with the same objective in 3D has been presented in Reniers et al. (2008), albeit based on a different approach than Katz’s.

Han et al. (2005) developed an alternative representation for the skeleton of a 3D object that overcomes the aforementioned difficulties. It is made from a host figure, a protrusion or indentation subfigure, and a description of the relation between the two. Its host skeleton is smooth and, intuitively speaking describes the host as if it did not have the protrusion or indentation, but it has a hole punched into its domain of (θ, *τ*_1_) into which the subfigure skeleton is smoothly attached. Its subfigure is either additive, for a protrusion, or subtractive, for an indentation, and it has a skeleton that has a fold at one end but which is truncated at the other end. Moreover, he devised a skeletal mechanism that attaches the truncated end of the subfigure skeleton smoothly into the hole in the host skeleton (Figures 8C-E). The attachment, which we call a “skirt,” represents the small part of the object boundary transitioning from the subfigure to the host. In the skirt the skeleton’s two sides separate from each other; as a result, the interior object region outside of the skirt is understood in the radial coordinates (*θ,τ*_1_,τ_2_), but the inside of the skirt contains no spokes and thus does not have radial coordinates. Despite this drawback, this context of s-reps with fitted frames began to be worked on. In that situation, the relation between the subfigure and the host figure can be expressed in terms of the fitted frames of the two figures’ s-reps. This setup has seemed attractive, but only recently has the continuation of this work on host figures and subfigures begun.

The surfaces of many manufactured objects have curves or points where the curvature is sharp: boxes are a good example. It would appear that there are natural skeletons of such objects, but the Blum skeleton will just not do. Far from the ends orthogonal to the longest axis of the box, the Blum skeleton stops being parallel to sides along the shortest axis and branches into long tracks accounting for the edge curves and corner points of the box. **What is needed is for the skeleton to run from end to end along the long and medium-length axis directions, and it must also have some sort of specialized protrusion marker to account for the edges and corners of the box. If such a description were to be invented, it would allow one to bring to bear all of the other beneficial aspects of shape analysis via skeletons**.

Another problem with manufactured objects is that many, at least according to their design, have the form of the non-generic entities that have circular symmetry, for example wheels and balls. The skeletons of such objects have a different dimension than general s-reps, so special steps would need to be taken to include these representations.

This concludes the description of the continuous s-rep. The following section describes the methods for fitting the discrete s-rep into an object described by its boundary.

## FITTING S-REPS TO SINGLE- AND MULTI-OBJECT BOUNDARIES

As introduced in Section Discrete S-reps, the discrete s-rep roughly is made from samples of the continuous s-rep, together with a mathematically appropriate means for interpolating the discrete s-rep into a continuous s-rep (Liu et al., 2021a). Typically, objects from images are represented by their boundaries or with binary images for which the cracks between within-object pixels or voxels and outside-of-object pixels or voxels form the object boundary. To utilize the benefits of s-reps, a means of fitting an s-rep to the object boundary is needed.

Why must the process of s-rep determination be a fitting to the input boundary and not a generation from the object boundary? Transformation of an object boundary to the skeleton has long been understood to be a process enhancing noise and detail. Despite hundreds of algorithms designed to accomplish this transformation, they have all foundered on the fact that little pimples or dimples in the boundary yield either a skeleton that is bushily branching or one for which the implied boundary of the resulting s-rep is unfortunately far from the object boundary that was input to the process. Methods for pruning the bush have been developed in 2D (Ogneiewicz and Kübler, 1995), albeit with a result that suffers from the inaccuracy problem just mentioned. And in 3D, despite many attempts by strong scientists, no adequate pruning method has been found. The result is that s-reps produced by generation from the boundary are poorly suited to statistical applications. The variable branching is what causes the problem.

So instead of going from the boundary to the skeleton, with its major enhancement of noise or detail in the boundary, the inverse operation of going from an s-rep to the input boundary has been shown to be successful, at least for certain objects, because it accomplishes a smoothing of the noise. The basic idea, as I see it, is to diffeomorphically deform the simplest skeletally described object into the target object and to carry the skeleton of the simply described object into the target object via that diffeomorphism. The simplest skeletally described object is the ellipsoid in 3D and the ellipse in 2D. The challenge then becomes determining what the diffeomorphism should be.

**FIGURE 9 ∣:**
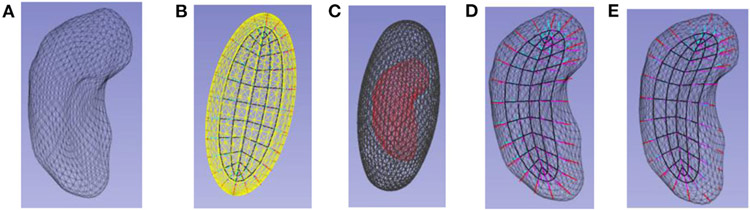
Fitting an s-rep into a hippocampus boundary. **(A)** The hippocampus, represented as a boundary mesh. **(B)** The ellipsoid, with its skeleton, into which **(C)** a hippocampus (red) is flowed. **(D)** The skeleton morphed into the hippocampus. **(E)** The refined s-rep.

**FIGURE 10 ∣:**
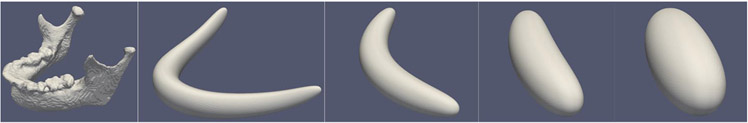
Conformalized mean curvature flow on a mandible.

The idea of fitting a skeleton (Liu et al., 2021a) to a target object boundary (see Figure 9) is to successively flow the boundary in an efficient way by smoothing it into an ellipsoid and then to reverse the flow while carrying the ellipsoid’s s-rep with it. The initial try at this flow (Hong et al., 2016; Hong, 2018) moved the boundary along boundary normals at a rate monotonic with the curvature at each (so far smoothed) boundary point. When the flow rate is proportional to mean curvature, it is unstable at regions of high curvature, but Kazhdan et al.’s (2012) conformalized mean curvature flow solves this problem (Figure 10). For almost all slabular objects of interest, this flow approaches an ellipsoid (an ellipse if in 2D), and one can check whether the smoothed boundary output from some iteration is close enough to an ellipsoid. The skeleton of that closest ellipsoid is analytically known. If that ellipse skeleton is deformed first to the approximate ellipse produced at the end of the flow, then the deformations provided by the smoothing iterations are successively reversed, and one has a diffeomorphism to the target object. This diffeomorphism can be applied to the skeletal and boundary ends of each of its discrete spokes, and the result is an s-rep for the target object. If the diffeomorphism does not correctly reflect skeletal properties (see item 1 in the next paragraph), the s-rep can be refined by a refinement diffeomorphism achieved by optimization of the following skeletal properties (Liu et al., 2021a):

1. foremost, a term heavily penalizing crossing of the spokes, via the comparison between boundary curvatures and the radial curvatures mentioned earlier;
2. a term penalizing the deviation of the implied boundary from the target object boundary;
3. a term penalizing the deviation of the angle of the spokes from the corresponding boundary normals.

**In addition, further closeness to mediality could be achieved by including a term penalizing the magnitude of the difference in the lengths of the two spokes emanating from the skeleton points that share a Euclidean location, and a penalty on the straightness of the spokes mapped from the ellipsoid**. The fitting algorithms found in the Slicer/SALT toolkit (Vicory et al., 2018) optimizes an objective function made from the first 3 terms, and at present it allows only the discrete spokes’ lengths and positions on the boundary to move, but **it probably would be effective to allow their positions on the skeleton also to move**.

Three things could be improved in this approach for producing geometric features in correspondence across a population:

1. The fitted frames and their locations depend on the diffeomorphism from an ellipsoid to each object in the population. But no intrinsic, fully satisfactory way to produce diffeomorphisms that works across a wide range of object shapes and yields correspondence across a population of similar shapes has been produced. Indeed, the s-rep that we produce is not as close to skeletal as is desired, as it maps straight spokes into curved ones. This prevents the fitted frames from meeting their goals as well as they could and makes correspondence across objects in a population that is needed for statistics less strong than it needs to be. Rather than having a refinement step, it seems that a better idea is to require the stages of backward transformation to keep the spokes straight and the velocities along them fixed in radial distances. Damon (2021) has shown how to do this when transforming one ellipsoid to another. In ongoing work in our laboratory we are attempting to generalize this to any slabular object by insisting that the sequence of points along the spokes stay straight.
2. The method of fitted frames produces correspondence to the extent that objects in a population are geometrically similar and the diffeomorphism computation for each reflect that similarity. Thus, the fitted frames for these objects reflect that similarity. However, the method does not explicitly reflect the statistical variation within the population as such, nor does it reflect any biological correspondences.
3. Following the human vision property reported in (Burbeck et al., 1996) that skeletal properties are measured at a spatial scale proportional to the object width (spoke length), the fitting could use this multi-scale approach.

Fitting a skeleton in 2D (Figure 3, right) operates in essentially the same way as in 3D, except that the model is an ellipse (Krishna et al., 2022). The skeleton being formed from an ellipsoid’s elliptical skeleton means that the curved skeleton formed by the diffeomorphism also has a spine and spokes, that is, the skeleton is also represented skeletally. Hong (2018) designed an extension of an s-rep for objects that can be understood to end in a cusp, such as the caudate nucleus. Vicory et al. (2022) has dealt with the problem of objects with multiple crests that must be put into biological correspondence by designing a diffeomorphism preceding the curvature-flow-based deformations, where the preceding flow smooths the high curvature regions in a way respecting their locations on the object within the members of its population. **A principled method for this preliminary analysis, based on the shape statistics of the boundaries in *the population* from which a particular object is a statistical sample, would be useful**.

## MULTI-OBJECT COMPLEXES

In many contexts, certainly in the human body, objects do not appear by themselves but in complexes of many objects. These objects can be separated or can share portions of their boundaries. In populations they can be adjacent or can pull apart, and they can slide along each other. The shape of an individual object is often correlated with that of nearby objects.

In our laboratory multi-object complexes in populations began to be studied as diffeomorphisms in a space of many objects, but with one object’s diffeomorphism related to its shape properties and also to neighboring objects’ shape properties (Saboo, 2011). However, we came to realize that richer geometric properties than just voxel (or pixel) positions were needed to describe the necessary relationships.

Multi-object and multi-figure fitting benefits from a representation that captures not only each of the component objects or figures but also the relations between them. Damon and Gasparovic (2017) has considered extension of the objects’ spokes into a “linking locus” where they meet, Liu et al. (2022) has implemented that scheme in a way that avoids folding. Krishna et al. (2022) considers objects that share a portion of their boundaries and uses a skeletal description of that shared region to capture inter-object information. This requires him to flatten that shared region before applying 2D s-rep fitting and then to restore the curvatures of the resulting 2D skeleton and spokes. He accomplishes that flattening by projection of the shared boundary region onto the skeleton of one (or both) of the objects and then projecting the skeleton back onto the ellipsoid’s medial ellipse whence it came. Before that, he modifies the 3D spokes of the two objects to be collinear in the shared boundary region so that deformations will resist interpenetration or pulling away from adjacency of the two objects.

Taheri and Schulz (2021) describes a different means of providing a linking locus, in which the linking is first described among the ellipsoids from which the object were diffeomorphically deformed and then the linking is transformed by a diffeomorphism common to the objects.

An attractive property of these multi-object representations is that the fitted frame can be extended from the interiors of the objects to the space between the objects. The early stages of doing this have been accomplished by Liu et al. (2022).

These multi-object representations appear to be useful not only for studying inter-class relationships but also for segmentation and, especially, segmentation editing, as described in Section Statistics on S-reps for Single and Multiple Objects.

## STATISTICS ON S-REPS FOR SINGLE AND MULTIPLE OBJECTS

The correspondence between spatial samples across an object’s training or testing population is critical to the success of the statistical operation. Because each s-rep for such a population is fit from a single shape and because the fitting is based on a rich set of shape features, s-reps do particularly well in producing correspondence. Tu et al. (2016) showed that even if the ultimate shape representation was a boundary PDM, choosing the spoke ends of a fitted s-rep as the boundary points yielded better statistical performance, at least on the objects she tested.

Because they have a heavy component of directional information and directions reside on unit spheres, the shape space on which s-reps reside is curved. One way to characterize an s-rep is as a tuple of n4 frames in 3D, a tuple of n3 3D directions, a tuple of n2 2D directions, a tuple of n+ positive variables such as lengths and an object size, and possibly a tuple of nL 3D locations. That is, an s-rep lives on (*S*^3^)^n4^ × (*S*^2^)^n3^ × (*S*^1^)^n2^ × (R^+^)^n+^ × (R^3^)^nL^. As discussed in detail in Pizer and Marron, 2017, each of the positive variables can be mapped from R^+^ to R^1^ (Euclideanized) using the logarithm and then mean centered by subtracting log(the geometric mean of the variable). The sphere-resident (directional) features need also to be Euclideanized before any of the standard statistical methods can be applied. We have accomplished those Euclideanizations using the Principal Nested Spheres method of Jung et al. (2012), which is a counterpart to Principal Component Analysis (PCA) for sphere-resident feature points. PNS is provided on the SlicerSALT toolkit (Vicory et al., 2018).

These representations for doing statistics require some sort of pre-alignment in position and orientation (and possibly spatial scale) to make the directions and locations consistent. Doing the alignment has the advantage that it can provide global object features for the statistics, such as its volume or the position of its center in a coordinate system based on some landmark. However, having to do the alignment generates difficulty because how to choose the alignment is unclear, even for a population of single one-figure objects. It is even more unclear for a population of multiple objects or multi-figure objects. The result is that the variability of the alignment adds noise to the representation and thus makes the statistical analysis less powerful.

As described in the following two subsections, Mohsen Taheri, Zhiyuan Liu, and Akash Krishna have used their s-rep fitted frames to generate s-rep features that do not depend on alignment. They have found these to provide particularly powerful hypothesis testing or classification on aspects of local geometry. This attractive idea has had only limited application because it is very recent, but I predict that it will become a method of choice quickly.

### Single Object Applications

Classification into s-rep classes simply uses s-rep features as the basis of classification, as initially studied in the work of Hong et al. (2016) on hippocampi between typical patients and first-episode schizophrenics and between hippocampi and caudate nuclei between typical 6-month olds and those who later developed symptoms of autism. He showed improvements in the classifications that used s-rep features over those that used only object boundary point features.

How many dimensions do the s-rep-based geometric properties involve? If the property is given by a fitted-frame or a rotation between a pair of frames, it lives on a hemisphere of a 3-dimensional sphere (understood to be embedded in 4 dimensions), so its representation requires a 3-tuple. Euclideanization of each of these thus yields 3 features. If the rotations of concern are with respect to positional changes in all three basis directions, that information requires a 9-tuple when Euclideanized.

If the geometric property is a direction related to a particular position, such as a spoke direction in an s-rep, it can be understood to live on its own 2-dimensional sphere representing directions in terms of a relevant frame and thus to require a duple when Euclideanized. However, it may be useful to understand the direction with respect to more than one coordinate frame, e.g., the frame at the skeletal end of an s-rep spoke and the frame at the boundary end of the spoke. In that example, a 4-tuple would be needed to express the direction.

If the geometric property is a point’s position, e.g., at some location in an s-rep’s skeleton, that position needs to be understood in some frame, e.g., of the s-rep’s center point, or even more than one frame. The position in each frame requires a 3-tuple. That 3-tuple can be expressed as a coordinate 3-tuple, or often more effectively as a direction (a duple) and a 1-dimensional, zero-mean Euclideanized length.

All of the aforementioned measurements can be made at many spatial scales to capture not only relations local to the s-rep but more global relations. After Euclideanization a PCA on the Euclideanized features is used to handle the covariance between the various curvatures, spokes, lengths, etc. Alternatively, as described in Sharma and Eltzner’s research (Sharma et al., 2021), if the feature-tuples cluster on the Cartesian product of spheres, they can be mapped onto a high-dimensional sphere, on which Euclideanization can take place, directly capturing the covariance between the various direction vectors. However, research so far has shown little advantage to the alternative method of Euclideanization in various classification applications, so our standard technique is sphere-by-sphere Euclideanization.

**FIGURE 11 ∣:**
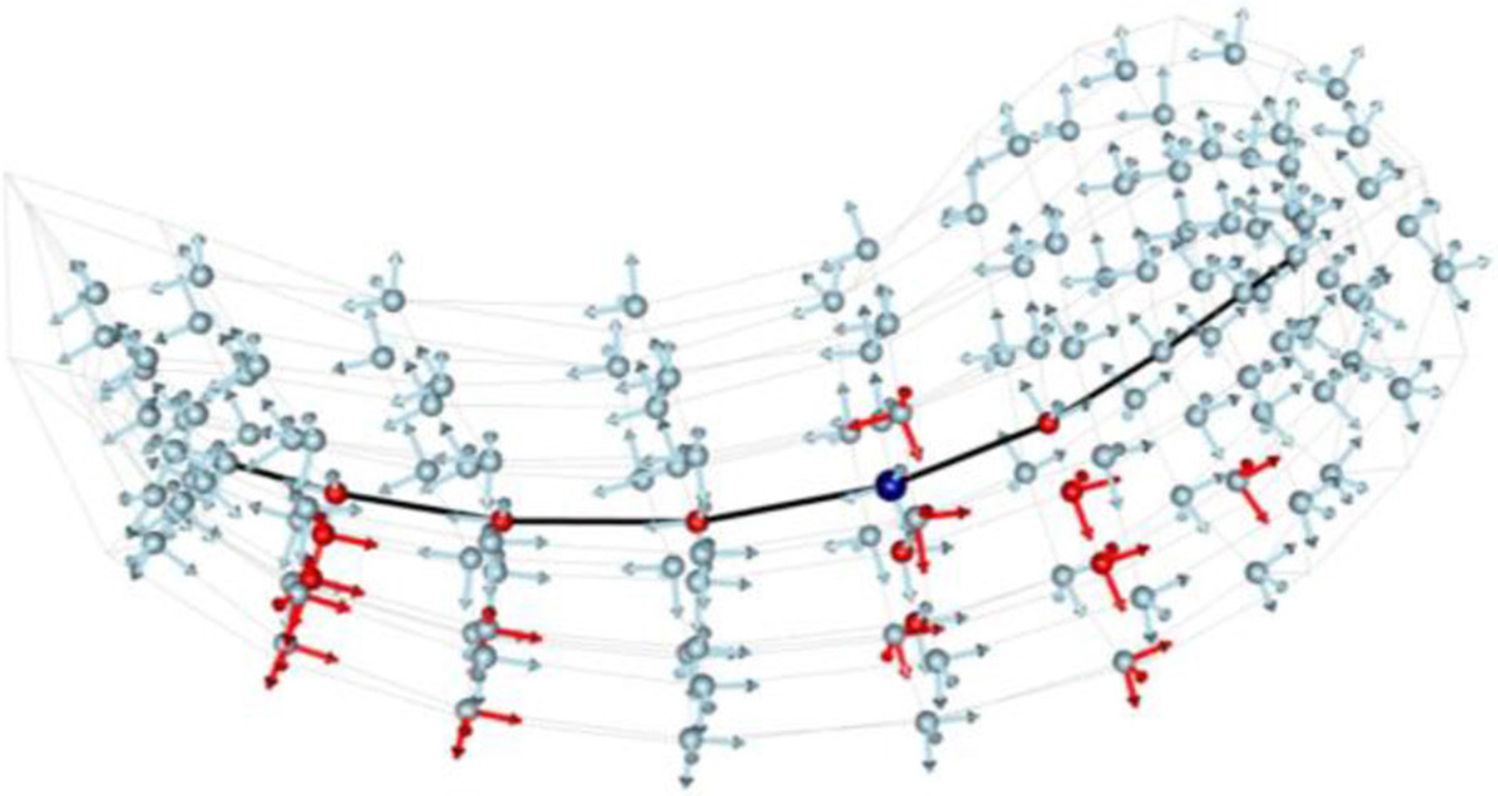
Hypothesis tests on hippocampi of Parkinson’s Disease vs. Control Group. Light-blue and red arrows indicate fitted frames representing non-significant and significant orientations, respectively. Light-blue and red spheres depict non-significant and significant positions. The dark-blue bullet is the center point. The black curve is the spine. Results are after *p*-value adjustment by Benjamini-Hochberg with False Discovery Rate (FDR) equal to 0.01.

Schulz et al. (2015) and Taheri and Schulz (2021) have used these features to do hypothesis testing between s-rep classes, studying which geometric object properties (feature tuples capturing a single geometric property, such as a spoke direction) differ significantly between the classes. Taheri showed in experiments comparing hippocampi between typical humans and those with Parkinson’s disease that the fitted-frame based features produce superior detections of differences than those using global coordinates. The introduced features, including point locations and frame orientations, were measured based on each fitted frame for either an object or a complex, with Euclideanization for the orientations. Correcting for multiple tests was accomplished via the moderate approach of Benjamini and Hochberg (1995). Taheri and Schulz compared the left hippocampi of 182 patients with early Parkinson’s disease (PD) vs. a healthy control group with 108 members. Figure 11 illustrates the result of the tests where red spheres and red arrows indicate significant positions and orientations, respectively. They saw four significant point locations on the spine. One may conclude that there is no bending or twisting as orientations are similar on the spine. However, a concentration of significant point locations and orientations at the lower middle part of the hippocampi may reflect a bending.

The boundary ends of an s-rep’s spokes imply a boundary Point Distribution Model (PDM). In a test data set of hippocampi Tu et al. (2016) showed that PDMs based on entropy-based correspondence of s-reps yielded better statistical properties than PDMs formed by entropy-based correspondence of the points themselves (Cates et al., 2006). Liu et al. (2021b) evaluated the jointly varying Euclideanized features from an s-rep implied multi-object PDM using the AJIVE method (Feng et al., 2018). He found that these jointly varying features produced better classifications of the hippocampus, caudate nucleus pair than the concatenation of the individual PDMs from the two objects.

Segmentation of objects from images requires knowledge both of the object shapes in the population and of the appearances in the image. Each of these can be represented by a probability density involving the object representation **z**: p(**z**), giving the shape information, and by the conditional density p(I ∣ **z**), where the elements of I are image intensity features, giving the appearance information. There has been quite a lot of research in our group on segmentations using s-rep features to make up **z** and producing the segmentation as the most probable **z** given I. When Bayes theorem is applied, it follows that the **z** resulting from this segmentation approach is arg max_z_ [-log p(**z**) + (-log p(I ∣ **z**))]. This approach was most heavily developed in the segmentation of organs in the male pelvis from CT for planning of radiation therapy of prostate cancer (Levy et al., 2007), and the methods were the basis of segmentation by a spinoff corporation, Morphormics (Holloway et al., 2008), which was later bought by Accuray. Vicory (2016) applied this notion to the segmentation of the prostate from 3D ultrasound, given its shape in MRI. That is, the probability densities needed were on shape change, as opposed to on shape. This required a normalization of the MRI shape in both the training cases and the target cases before the statistics could be computed or used. This normalization was accomplished by applying a mean s-rep deformation to the s-rep from the patient’s MRI before finding the shape change with the maximum posterior.

In Vicory’s work the intensity features were not only image intensities but also derived texture features. Also, the appearance log probability was based on probability densities giving the probability of a voxel being inside the object given the intensity and textures tuple.

Certain objects produced by automatic segmentation methods need editing via user interaction. When the editing is done for a 3D object, editing the image slice by slice is too time-consuming. Thus, segmentation editing is an important objective. We believe that this editing should combine some but limited user specification on image slices, geometric information from acceptable segmentations in image slices, and image appearance information. Mostapha et al. (2017) built a system based on s-rep statistics on shape changes needed, but it did not include a basis on appearance information. **Also needed would be s-rep-based shape changes of neighboring objects conditioned on the changes of the prime objects. That work is yet to be done**.

#### Evaluations via Statistics

Since s-reps were designed to produce features useful for statistics on shape and in particular to produce spatial and orientational correspondences needed for statistics on populations of shapes, the evaluations need to be according to measurements of statistical success. The chapters (Pizer and Marron, 2017; Pizer et al., 2019) give many such measurements. In summary, on all of the anatomic objects tried and both of the diseases investigated for shape effects, the s-rep features and the methods of nonlinear statistics produced superior classification and hypothesis testing to the more traditional methods based on boundary points. Also, measures of generalization and specificity of the derived probability distributions led to preference for s-reps. Whether these preferences would follow through for features based on diffeomorphisms in a containing space has not yet been investigated, but the fact that object width features have been shown to be important for good statistics and these features are not directly available from diffeomorphisms suggest that even there statistics via s-reps might prevail.

The next section gives some recent results showing that statistics based on multi-object features also has strengths relative to alternative models.

### Multi-Object Statistics Using Skeletal and Boundary Fitted Frames

A particular capability of s-reps is how it enables powerful statistics on multiple objects. We present two approaches that have been developed.

#### Classification and Hypothesis Testing on Separated Objects

**FIGURE 12 ∣:**
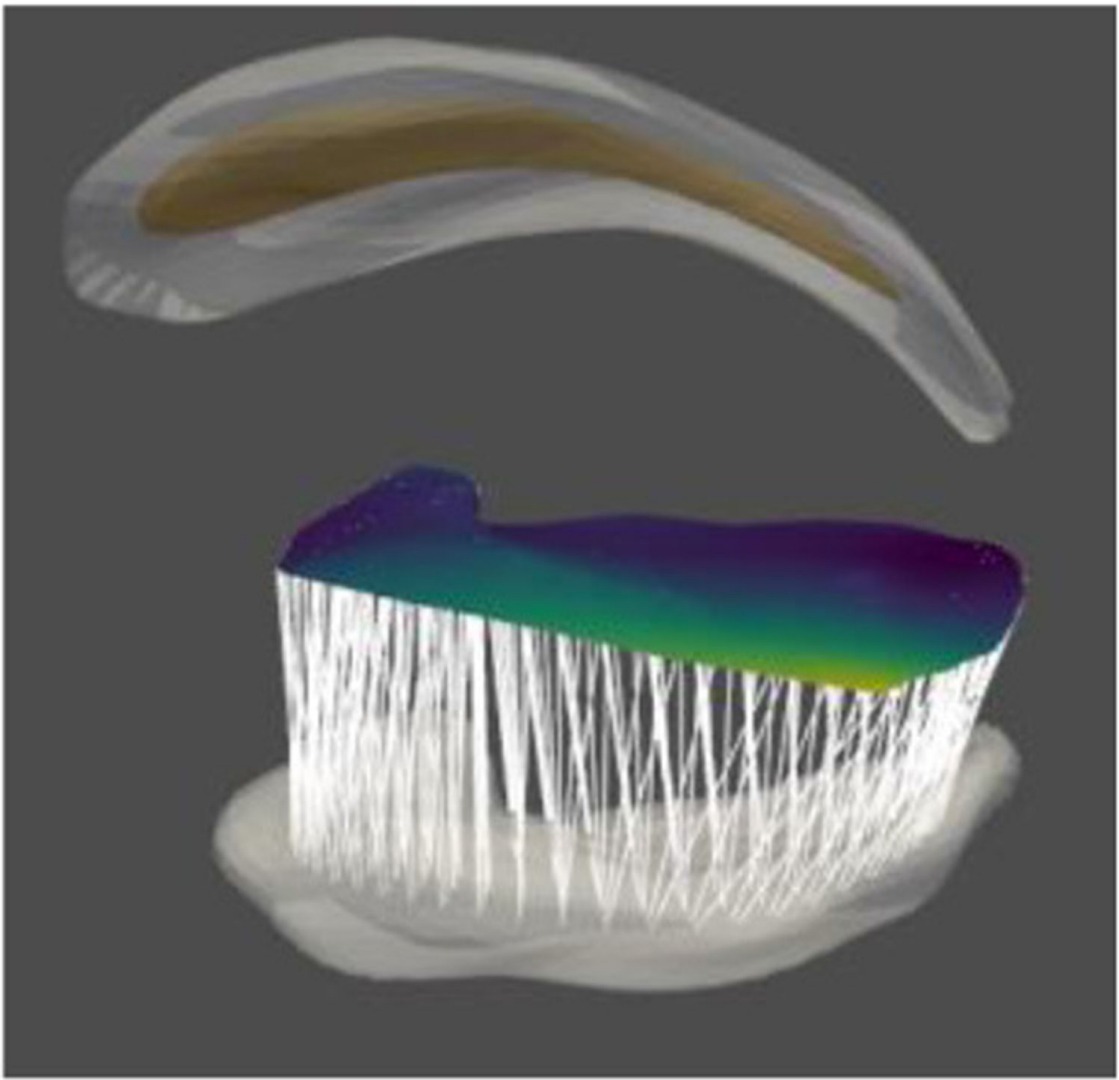
An infant’s hippocampus (surface shown at the bottom); the caudate nucleus, shown at the top by its boundary and skeleton; the inter-object linking surface in the middle colored by link length from the hippocampus; and the discrete links from the hippocampus shown for each discrete hippocampus spoke.

Zhiyuan Liu has completed dissertation research (Liu, 2022) using data from two separated subcortical brain objects in infants, as imaged by MRI: the hippocampus and the caudate nucleus (Figure 12). The data fall into two classes related to whether the child will develop autism. He has developed multiple geometric features that can be used in classifications into the two classes, as well as hypothesis testing on the relation of features to classes. On these bases he has compared the use of various s-rep-related features. The results suggest that the following aspects are especially important:

Multi-object features that include between-object linking information, combined with within-object features classify more strongly than just within-object features.

- S-rep based features are particularly powerful, and especially affine frames produced by mapping s-rep fitted frames from the base ellipsoid to the target object.
- A desired set of between-object features are lengths and directions of link vectors extending s-rep spokes in a 1-to-1 fashion to a linking surface between the objects.
- Focusing on features varying jointly as an effect of a disease provides advantage over features selected without regard to joint variation.

These conclusions are also presented in two papers (Liu et al., 2021b, 2022). The first of these describes a method called NEUJIVE in which Euclideanized multi-object features are analyzed into joint features using the statistical method called AJIVE (Feng et al., 2018). The second describes how representing between-object shape using links from one object to a linking surface between objects provides superior classification and hypothesis testing.

Here we briefly detail both the definitions and use of affine frames and of the linking vectors. The triplet of vectors in each affine frame are no longer unit length nor need they be orthogonal. They allow avoidance of preliminary alignment, which is especially challenging for multi-object complexes. They allow locations to be understood in the coordinate system of an object’s skeletal center point. As well, the relation of each affine frame to that at that skeletal center point captures shape information itself.

The linking surface is formed by a use of Damon (2021)’s linking mathematics. It smoothly and bijectively interpolates landmark pairs from equal length spoke extensions from the two objects’ surfaces where there is no folding due to within-object spoke intersections. The links from an object to that surface are thereby smoothly interpolated to connect each discrete spoke to the linking surface (Figure 12), thereby depending on the good correspondence properties of discrete s-reps.

However, what the best features are to describe inter-object relations is still an open question.

#### Classification of Abutting Objects

**FIGURE 13 ∣:**
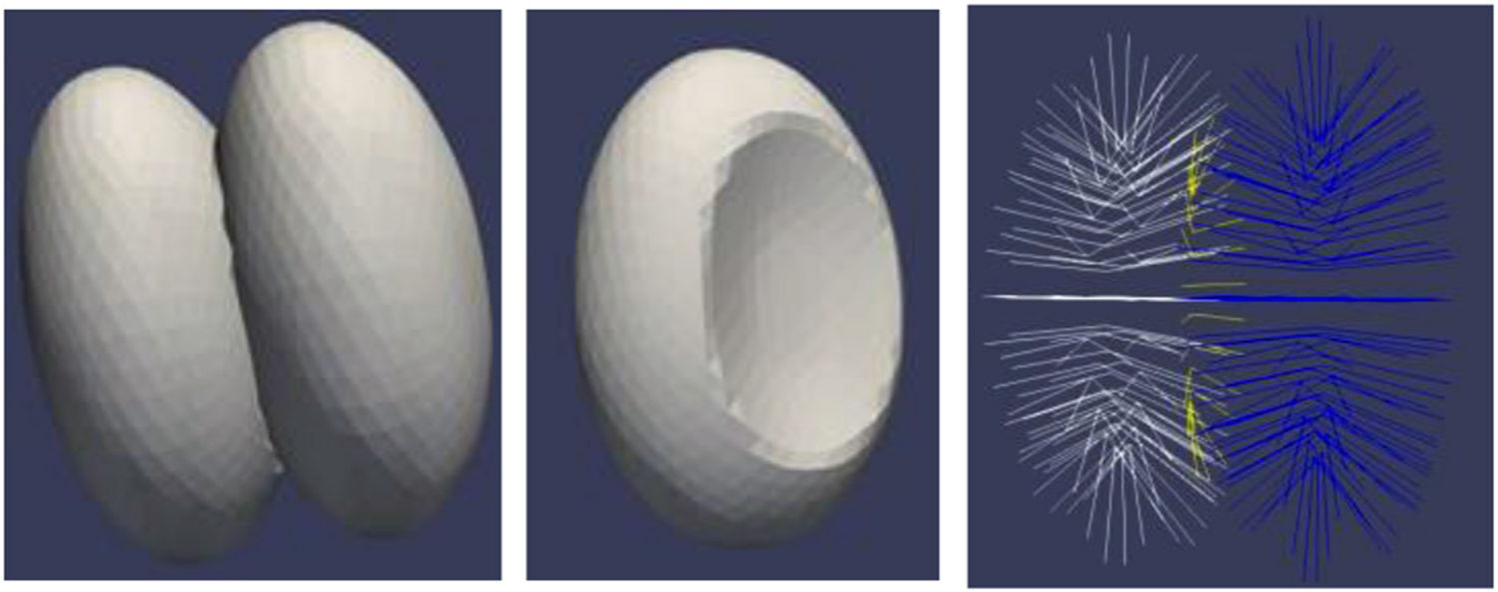
Ellipsoidally based objects with a shared boundary that are used as data. Left: The two objects. Middle: one of the objects, showing the region of shared boundary. Right: the 2 object s-reps and the shared boundary s-rep (yellow).

Krishna et al. (2022) has work developing the ability to represent two objects with parts of their boundaries shared (Figure 13). His method involves providing s-reps of the two objects, where those s-reps’ spokes are collinear within the shared boundary region. Moreover, he computes an s-rep of the 2D shared boundary as well. Not only are the features of the two objects understood in terms of their own fitted frames, but the s-reps features of the shared boundary region are understood in the fitted frames of one of the two objects.

Krishna compared classifications on deformed ellipsoid pairs sharing a boundary region. One experiment measured the accuracy of classifying a pair of deformed ellipsoids where one ellipsoid is bent from a pair of ellipsoids where neither object is bent. The other experiment measured the accuracy of classifying a pair of deformed ellipsoids where one ellipsoid is stretched relative to the other from a pair of ellipsoids where neither object is stretched. These classification capabilities were compared between s-rep features, using fitted frames, that ignored the shared boundary region’s geometry vs. ones that included that geometry.

The number of s-rep points used was the same in all of the comparisons. His results show the benefits of inclusion of the shared boundary’s s-rep features for object bending but not for object stretching. He also began work on a pair of brain structures that share part of their boundary. It did not adequately find a way to produce a smooth region of shared boundary from objects segmentations with individual segmentations in the form of coarse triangular tiles. **Future work should analyze brain structures having shared boundaries with such a technique. Also, a method using Liu’s linking surfaces that include the shared boundary would be worthy of development**.

## DIFFICULTIES WITH AND LIMITATIONS OF SKELETAL REPRESENTATIONS

The skeleton of a 3D object is most well understood when the principal radii of the ellipsoid generating that skeleton are all notably different from each other. A particular problem is populations within which in one part of the population the longest object axis corresponds to the second longest axis in another part of the population or the population contains objects for which moving along the longest axis makes the second longer axis transition to be shorter than the one that was third longest—the transition is generic even though the transition shape, with a circular cross-section, is not. Moreover, when the smaller two of the principal radii remain close for an interval along the longest axis, the resulting near-circular symmetry makes the skeletal surface very thin and the orientation of the skeleton unstable. When this happens for objects considered as a quasi-tube, the skeletal orientation about the tubular axis (the spine) will seem to discontinuously change. **Work to deal with this behavior is needed**.

A strength of s-reps is that they are insensitive to noise in the boundary that yields pimples and dimples. Yet in some applications, e.g., where two objects must fit together tightly to form a seal against fluid leakage, the boundary must be expressed in a form that has no noise, e.g., with boundary intervals specified by splines. This is a real advantage of cm-reps (Yushkevich et al., 2015), where the implied or explicit spokes are normal to the boundary. **A challenge is to create a form of s-reps where subregions are restricted to having spokes normal to the boundary**.

While forms of fixed branching of at most a few levels could be easily handled by s-reps and their statistics, variable branching, such as happens in most tubular trees in the body, would require statistics of branching. Methods of the statistics of branching is a somewhat immature discipline, and its application to s-reps has not been accomplished.

In the simplest situations the fold of the skeleton in an s-rep is opposite a crest of the boundary. However, on the boundary’s crest region, along a principal curve crossing the crest, the zero level curve of the derivative of principal curvature can transition into an undulation with two crests and a trough. **In that case how the skeletal fold should behave has not been understood, to my knowledge**.

The major limitation of s-reps as the basis for statistics is that the data is typically provided as a mesh of triangular tiles representing the object boundary and that the s-rep must be fitted to that mesh. This weakness is shared with the many valuable methods for statistics based on computing a diffeomorphism over space including an object and then doing statistics on features derived from that diffeomorphism (see, for example, the Deformetrica library; Durrleman et al., 2014). An interesting alternative is the recently published work (Ambellan et al., 2021) that does its statistics directly on deformations of the mesh itself. **Research comparing these various methods for doing statistics on shapes would be valuable**.

Other limitations come from the fact that when corners and sharp edges are important features, as they are in manufactured objects, s-reps at present need to treat those somewhat unnaturally as subfigures. They also handle randomly branching objects such as blood vessel trees poorly, as well as objects in a single population that are in different topological classes or have different arrangements of their subfigures.

## DISCUSSION AND CONCLUSIONS

### Potential Nonmedical Applications

The chapter by Leymarie and Kimia (2008) lists a large number of applications to which skeletal models have been applied, from the cosmological scale to the atomic scale. **However, none of those used s-reps, but they could.** The following discusses applications where s-reps could have been used, and it discusses extensions of those as well as new opportunities for non-medical applications.

Designing objects for manufacturing or art often nicely involves combining figures that can be understood skeletally. Means with an intuitive interface for specifying the shape of single figures using a limited set of primitives have already been worked on (Wikipedia^3^). However, s-reps would provide a much wider range of shapes as primitives. Means for controlling the way figures are pasted together would need to be invented, where these means were adequately intuitively controllable by the designer.

Computer graphics and the subset of that field toward visualization also could benefit from a wider set of primitives than are presently routinely used.

The human body is made from articulated figures. Each figure, e.g., the forearm, has a recognizable shape, which can be statistically studied, not only statically but also in motion. While there are many studies of the body in motion as stick figures, it would seem helpful to study the body with flesh on its bones using the s-rep’s efficient features. The ideas of figure-to-figure connections described above would appear to be useful here. Similarly, robots and other mechanical devices made from articulated figures, such as airplanes, could benefit from using s-reps.

**FIGURE 14 ∣:**
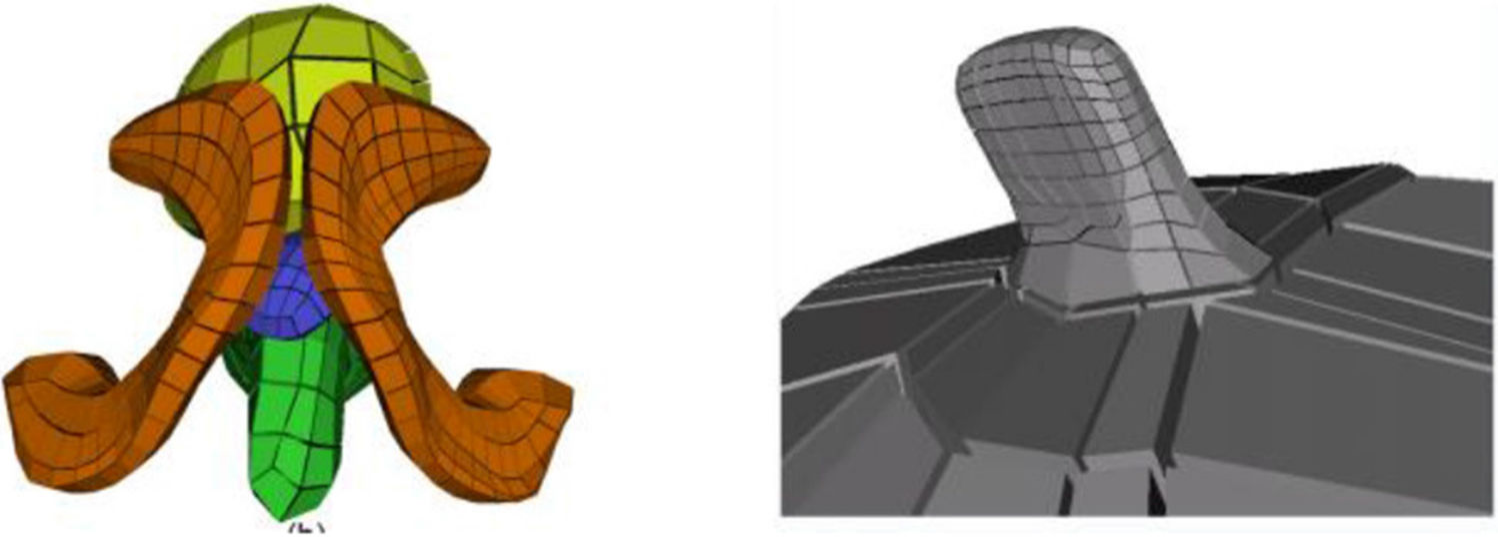
Left: Finite elements for objects in the male pelvis. Right: finite elements for a host figure and subfigure (Crouch et al., 2007).

Once we have mechanical models formed from skeletons, it is natural to consider mechanical motion of these models. And this does not need to be restricted to articulation. Crouch et al. (2007) showed how skeletal models (albeit not s-reps) could be used to divide an object or a group of objects into natural elements for multi-scale finite element modeling of mechanical changes (Figure 14). For a single-figure object the subdivisions were along *τ*_2_ and θ. For multi-figure models including protrusions she showed how to have the finite elements transition from the subfigure elements to the host figure elements, providing an alternative to Han’s approach described above. **Certainly, the shape properties revealed by a skeletal model make further work on physically based modeling using s-rep based elements an attractive direction**.

While s-reps have been used only for data that was extracted or is being extracted from medical images or images from ordinary cameras, its application to data extracted or being extracted from other sensors would be useful. For example, the LIDAR sensors used in self-driving vehicles would be an interesting source.

Human vision certainly divides the world into objects. Biederman (1987) and Burbeck et al. (1996) (in our group), and many others have adduced psychophysical evidence that it does so via skeletal primitives, which Biederman calls “geons.” Burbeck and Pizer also gave evidence that the human visual system’s skeletal analysis was done at spatial scales proportional to the object width. Indeed, there is some limited evidence that the monocular visual system is especially sensitive at skeletal points (Lee, 1995; Lee et al., 1998). Without some direct way for the brain to sense objects, how else could it be so fast in such sensing? Moreover, we have a good sense for categories of objects, e.g., faces and trees and roads. The instances within these classes differ geometrically from each other, but they typically have similar skeletal topology. **It seems natural to study mental models used in recognition by using s-reps**.

### Desirable Future Research on S-Rep Methodology

So far, when regions of the skeleton have been considered, they have been limited to ones entirely on either the north side or the south side of the skeleton. However, regions folded on a skeleton arise, for example, when an object abuts another across a crest into which the skeleton fits. It should be straightforward to include such regions as a possibility.

So far, s-reps have been created only for single-figure or multi-figure slabular objects, i.e., those with spherical topology and have a skeletal surface, or for generalized cylinders, which also have spherical topology and focus especially on a curvilinear skeleton (Saboo, 2011). But there are many other topologies for which a skeletal model is appropriate. Cyclic forms of skeletal surfaces, such as closed fists, or of skeletal curves, for example, of doughnuts could be very useful. Likewise, s-reps for annular solids, such the myocardium would be useful.

In some populations a pair of objects in some instances share a boundary and in others the two objects are separated. It can even happen that one of the objects melds with the other object becoming a subfigure. Statistical methods for such populations could be developed. Also, one might want to be able to handle a host figure with two subfigures that can in some instances touch and in others be separated, and even meld together in such a way to change the number of holes (topological index).

Other situations needing development come when there are more than two objects. Handling how one object slides along the other two within the population could be handled via Liu’s linking surfaces (Liu et al., 2022), which uses fitted frames. Also, the abutment arrangements can vary across cases, e.g., as one of the objects slides along the other. Cardiac valves can present such distributions.

Multi-scale s-reps would be worthy of study. For example, taking the spine as a whole at one spatial scale, without reflecting the shapes of the individual verterbrae, and then describing the vertebrae at a smaller scale, and the vertebral parts at a yet smaller scale would provide a driving problem.

Objects can be in motion. Especially when the motion involves deformation, as in the beating heart or breathing lung, statistical analysis of the motion sequences is of interest. Hong et al. (2019) has studied the progression of Huntington’s disease statistically using cm-reps, Yushkevich et al. (2015) has studied objects in motion using his cm-reps, but such studies could usefully be extended to s-reps because the fitted frames will give powerful ways of characterizing the deformation. In general, comparisons between s-reps and cm-reps for a variety of applications would be informative.

It seems straightforward to apply this s-rep idea to higher-dimensional objects as long as they have codimension 1; i.e., where the ambient dimension is some n and the object boundary has dimension n-1. Far from straightforward, but likely important, would be the extension to a number of spatial dimensions and time. The difficulty is that a metric in (space, time) is complex because space and time are incommensurate. Making them commensurate would seem to require ideas of relativity. As exciting as this would be, it is beyond the scope of short term research, I believe.

Because the geometry of s-reps involves frames, which live abstractly in SO(3) (a hemisphere of *S*^3^), and directions, which live abstractly in *S*^2^, the space describing a whole discrete s-rep lives on a Cartesian product of spheres, i.e., a polysphere: (*R*^+^)^*d*1^× (*S*^2^)^*d*2^× (*S*^3^)^*d*3^, where *d2* counts the number of vector directions and *d3* counts the number of frame directions. The PNS algorithm for doing a PCA-like dimension reduction (Sharma et al., 2021) can be applied sphere by sphere, but it leaves the question of how to handle polyspheres that are more toroidal. That is, research on how from points on a toroidal polysphere of dimension *d* to create a subdimensional surface of dimension *d-1* that best fits the points is needed. Such research has recently been reported (Zoubouloglou, 2021) on Cartesian products of 1-dimensional spheres (circles), and **extension on the Cartesian product of 2-dimensional spheres is anticipated, but extensions to polyspheres made of 1-, 2-, and 3-dimensional spheres is needed**.

Good positional and orientational correspondence among instances of an object in a population is particularly important for statistical applications. Information as to this correspondence can come from understanding the objects in their source environment, e.g., biological correspondence for anatomical objects or structural correspondence for manufactured items. Or they can come from entropy analysis (Davies et al., 2001; Cates et al., 2006; Tu et al., 2016), though this can be very time-consuming and subject to local optima in optimization schemes. Or they can come from deep learning. **Methods for improving the correspondence by such means would probably be quite helpful to statistical or deep learning applications**.

On the subject of deep learning for operations related to objects, such as recognition or segmentation, there is the open question of whether deep learners that are based on largely linear operators (other than the ReLus) on base elements such as voxel values or mesh node locations and links can compete with deep learners that use as features s-rep frames and connecting vectors, which are nonlinearly related to those more basic features.

## CONCLUDING REMARKS

While not every object of study is suitable for representation by s-reps, there are many that are and that appear to be especially suitable for statistical analysis. This is because 1) object shape is best understood through a population of objects; 2) the s-rep methods presented here have notable advantages in providing descriptive object features with correspondence across the population, indicated by studies on anatomic objects. **While many of the theoretical challenges in developing and using s-reps have been met, there are many more remaining, and application opportunities abound**. The software in the SALT shape analysis toolkit (Vicory et al., 2018) supporting s-reps already can provide help to developments and uses of s-reps, and more will be forthcoming from my laboratory. **Especially, bringing the advantages of s-reps to manufactured objects and to design remain an open challenge**. I hope that this paper will encourage others to take up this work. They will be welcome to add their software to the SALT s-reps collection.

## Data Availability

Inquiries on the data used in the many studies reported in this paper can be directed to the corresponding author. Much of this data is no longer available, and other of it is owned by other groups and is not publicly available.

## Footnotes

1 In this paper “I” refers to concepts and approaches specifically credited to the first author, while “we” refers to the collection of authors.

2 Many of the figures in this paper also appear in Pizer et al. (2021).

3 Wikipedia on 3D sculpting. Available online at: https://en.wikipedia.org/wiki/Geometric_primitive.
